# Mistriaged Advanced Life Support Patients in a Two-Tiered, Suburban Emergency Medical Services System

**DOI:** 10.5811/westjem.2019.10.43885

**Published:** 2020-02-24

**Authors:** Joshua Bucher, David Feldman, Joslyn Joseph

**Affiliations:** * Rutgers - Robert Wood Johnson Medical School, Department of Emergency Medicine, New Brunswick, New Jersey; † RWJ Barnabas Mobile Health Services, New Brunswick, New Jersey; ‡ Morristown Medical Center, Department of Emergency Medicine, Morristown, New Jersey; § Newark Beth Israel Medical Center, Department of Emergency Medicine, Newark, New Jersey

## Abstract

**Introduction:**

Emergency medical services (EMS) systems exist to provide prehospital care in diverse environments throughout the world. Advanced Life Support (ALS) services can provide advanced care including 12-lead electrocardiogram (ECG), endotracheal intubation and parenteral medication administration. Basic Life Support (BLS) can provide basic care such as splinting, wound care and cardiopulmonary resuscitation. ALS can release patients to BLS for transport to the hospital, and this is an area of high risk. Our study examines patients who were triaged and admitted to a critical care location, including an intensive care unit (ICU), cardiac catheterization laboratory, or operating room (OR).

**Methods:**

The analysis included data from 2007–2015 of all patients who were triaged. We evaluated demographics, admission diagnoses, and dispositions using descriptive statistics. Diagnoses were grouped into categories based on the system.

**Results:**

We found that 372/17,639 (2%) of patients were mistriaged to BLS and admitted to a critical care location. The average age was 64. The most common diagnosis categories were neurological (24%), gastrointestinal (GI)/abdominal pain (15%), respiratory (12%), and cardiac (12%).

**Conclusion:**

It is uncommon for patients triaged from ALS to BLS to be admitted to an ICU, catheterization lab or OR, with a rate of 2%. Neurological, GI, respiratory, and cardiac diagnoses were the most frequent categories of patient complaints that were mistriaged. This study should lead to further studies to examine this patient population.

## INTRODUCTION

Worldwide, many different emergency medical services (EMS) systems exist in order to serve diverse patient populations. One of the systems in the United States uses a two-tiered response comprised primarily of a Basic Life Support (BLS) transport ambulance staffed by emergency medical technicians (EMT) and Advanced Life Support (ALS) staffed by two paramedics. A tiered system has the advantage of spreading resources further by incorporating volunteer, public, and private BLS ambulances. With more ambulances available to respond to simultaneous patients in high-volume areas, response times to critical intervention such as cardiopulmonary resuscitation and stabilization of trauma patients may be decreased.^1^ One challenge of EMS is determining which patients truly require ALS pre-hospital care. An emergency medical dispatch (EMD) protocol will automatically dispatch ALS units to high-acuity complaints, such as chest pain, shortness of breath, altered mental status, and trauma as specified by protocols. EMD protocols decrease inappropriate dispatches of ALS in cases where advanced medical procedures and interventions, such as intravenous (IV) access, fluid resuscitation, medications, or cardiac monitoring are not necessary.^2^ ALS interventions have shown to provide some mortality benefit to patients with acute myocardial infarction, in certain trauma patients, and for seizures.^3^,^4^,^5^

An ALS unit that is dispatched and responds to a scene may down-triage or “release” the patient to BLS if, after ALS assessment, the paramedics feel no ALS monitoring or interventions are warranted. This process is either done through standing protocols or consulting an emergency physician (EP) via online medical control. This is an accepted practice in EMS systems that operate in a tiered-response environment. To date, no studies have been conducted to evaluate this group of patients who are triaged to BLS and subsequently found to have a condition requiring admission to the operating room (OR), cardiac catheterization lab, or intensive care unit (ICU). These groups will be referred to as a critical care location. Cardiac monitoring is required for patients who are admitted to critical care locations; this would suggest there may be benefit to ALS monitoring, treatment, and transport to the hospital. This is a high-risk group that may warrant ALS intervention and represent an area of opportunity to improve.

In this study, we sought to characterize a sample of cases where mistriage from ALS to BLS occurred. We used a large, suburban, hospital-based EMS agency with consecutive patients using a protocolized, retrospective chart-based review.

## METHODS

The setting is a suburban, two-tiered EMS system in which ALS units evaluate approximately 14,000 patients per year. Patient charts are documented in EMSCharts (Zoll Medical, Chelmsford, MA), a commercially available electronic medical record (EMR) designed for prehospital care. Inclusion criteria were cases mistriaged – patients who were triaged from ALS to BLS and admitted to an ICU, cardiac catheterization lab, or operating room from the emergency department (ED) (critical care locations). For the analysis, we retrospectively reviewed data on all patients from 2007–2015 who were down-triaged to BLS, transported to an ED, and then were subsequently admitted to a critical care location. From this group, demographics, diagnosis category, and disposition were extracted via EMSCharts into a spreadsheet that was analyzed for descriptive statistics using Excel (Microsoft Corp, Redmond, WA). We calculated 95% confidence intervals when appropriate. We excluded from analysis patients who were triaged to BLS for transport and not admitted to an ICU, OR, or catheterization lab.

The disposition of patients was obtained by the individual paramedic, EMT, or supervisor and was documented in the patient chart. Diagnoses were recorded and subsequently classified into categories that were programmed into the EMR and selected by the individual who obtained follow-up information. This study was approved based on a universal institutional review board (IRB) approval for retrospective chart reviews of EMSCharts data granted by the IRB at Morristown Medical Center in Morristown, New Jersey.

**Population Health Research Capsule**

What do we already know about this issue?

In a two-tiered emergency medical services (EMS) system, many patients are often mistriaged despite having life threatening diagnoses. No studies have previously characterized this phenomenon.

What was the research question?

What are the characteristics, diagnoses, and dispositions of patients who were mistriaged from Advanced Life Support to Basic Life Support?

What was the major finding of the study?

The mistriage rate is 2%. Patients are often geriatric. Neurologic, gastrointestinal/abdominal, and sepsis diagnoses were most often missed.

How does this improve population health?

Focusing paramedic education on recognizing these frequently missed emergencies may lead to safer triages and management of prehospital patients in two-tiered EMS systems.

## RESULTS

**Figure 1:**
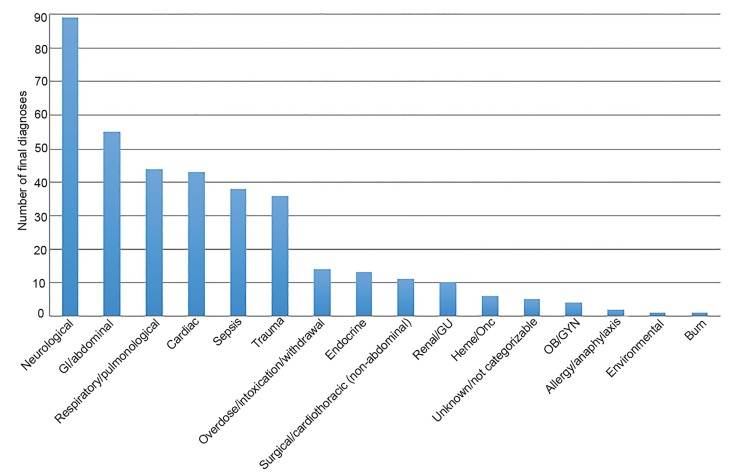
Number of admission diagnosis categories for patients triaged to basic life support and admitted to critical care location. *GI*, gastrointestinal; *GU*, genitourinary; *Heme/Onc*, hematology/oncology; *OB/GYN*, obstretics/gynecology.

**Figure 3:**
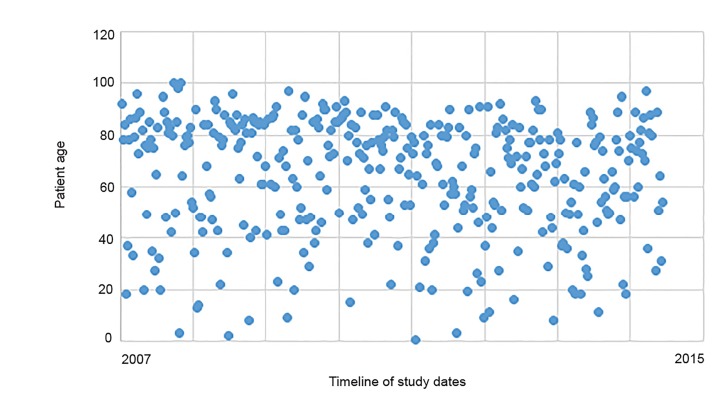
Scatter plot of ages of patients triaged to basic life support and admitted to critical care unit.

Out of 17,639 patients from 2007–2015 who were evaluated by ALS and triaged to BLS, 372 patients (2%) were mistriaged to BLS. The average age was 64 years, and 52% were female. The most common mistriaged admission diagnosis category was neurological (24%), followed by gastrointestinal (GI)/abdominal emergencies (15%), respiratory (12%), cardiac (12%) sepsis (10%), and trauma (10%). Of patients who were admitted, 83% went to an ICU, 15% to the OR, and 2% to the catheterization lab. Please refer to Figures 1, 2A, 2B, and 3 for the full results.

## DISCUSSION

This study, while limited, demonstrates several important concepts. Our study demonstrated that there was a 2% rate of mistriage to BLS. These are critically ill patients who require close monitoring and could potentially benefit from ALS interventions and support that cannot be provided by EMTs. In patients who are admitted to the OR, ICU, or catheterization lab, cardiac monitoring is standard care. At the bare minimum, this data demonstrates a missed opportunity to closely monitor the patient for deterioration. In the state of New Jersey, all cases of identified mistriage have to be reported to the Department of Health Office of Emergency Medical Services.

The average age of patients mistriaged was 64, and the median age was 70. The most frequently missed complaints included neurological and GI/abdominal complaints. Older patients with complaints of abdominal pain have more frequent and more serious diagnoses than younger cohorts.^6^ Likewise, neurologic complaints were also frequently missed. Although the specific chief complaints were not analyzed, it is possible that patients may have presented with vague complaints, such as “dizziness, headache, fatigue or weakness.” This knowledge could influence EMS education in that more caution should be taken when considering older patients for ALS transport with vague complaints who may become ill.

Patients admitted to the OR represent a significant area for improvement; it is possible that ALS providers are not recognizing situations in which emergency surgery may be indicated. This has serious implications for prehospital. It is possible that preoperative patients who may benefit from IV access, fluid resuscitation, pain and nausea medication may not be receiving it as a result of triaging to BLS providers. This is a group that requires further study, as patients may be admitted to the OR with non-critical diagnoses and straightforward surgeries that may not necessitate ALS care.

Lastly, 2% of patients were admitted directly to the catheterization lab. In the era of paramedic interpretation and 12-lead transmission of electrocardiograms (ECG) directly to the ED or to the catheterization lab, this is a population in which there should be few misses. However, it is possible that not all these patients met ST-elevation myocardial infarction (STEMI) criteria, and that some patients were taken to the catheterization lab based on dynamic ECG changes in the ED or for other reasons. It is also possible that the initial ECG may have had signs of a non-STEMI (NSTEMI) or STEMI and were simply misinterpreted. Either way, these were patients whose disposition implied they required a higher level of care than BLS and represent an area for improvement.

Our EMS agency had a protocol to guide paramedics in decision-making for down-triage to BLS providers for transport. However, it is possible that the protocols were not followed. In that case, education is required for the providers who violate them, but there is no ability to override their decision-making in real time.

## LIMITATIONS

There are several limitations worth mentioning. This is a single EMS system study with a largely suburban response area in New Jersey. These results may not be generalizable to systems of dissimilar characteristics. The state of New Jersey operates a two-tiered system with BLS and ALS in separate ambulances, and ALS ambulances are staffed with two paramedics. Paramedics cannot transport in New Jersey under most circumstances and require BLS to transport the patient. This has the potential to influence decision- making.

We only examined data from patients who were admitted to critical care locations after ALS had triaged them to BLS. We did not compare this data with patients admitted to the respective units without triaging or patients treated by ALS and who were then admitted to these locations. Therefore, conclusions are limited to descriptions only. Disposition of the patient was determined by either in-person follow-up in the ED or by phone. It is possible patient’s dispositions may have been missed. Likewise, patients in whom disposition was unable to be determined, or disposition was not investigated, were not included in this study. Disposition may have been mistaken. This may have skewed the results. Different hospitals may have different criteria for admission. This can also affect the results.

Individual paramedics may have variation in their rates of triage. This has important implications for the performance improvement process to identify special cause variation in individual departments. Lastly, due to our study design, we did not have a control group to make statistical determinations nor did we have patient outcome data, such as disposition status from the hospital. This also limits the conclusions that can be made to descriptions of this population.

## CONCLUSION

This is the first study to investigate mistriaged patients from ALS to BLS. The data may help guide system planning and direct future research efforts to improve clinical care regarding patient triages. Future studies should include reviewing the outcomes of mistriaged patients to determine which of these patients, if any, suffered poor outcomes potentially related to mistriage and include a control group. We believe that further research on this topic is important in order to make safe decisions for prehospital patients and continue to use resources in two-tiered EMS systems effectively.

## Figures and Tables

**Figure 2A:**
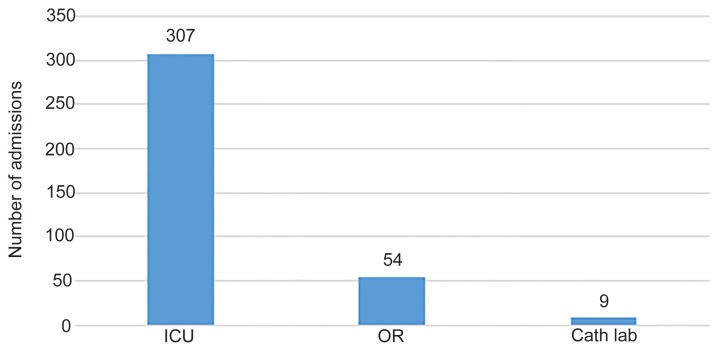
Admission critical care location of mistriaged patients. *ICU*, intensive care unit; *OR*, operating room; *Cath Lab*, catheterization lab.

**Figure 2B:**
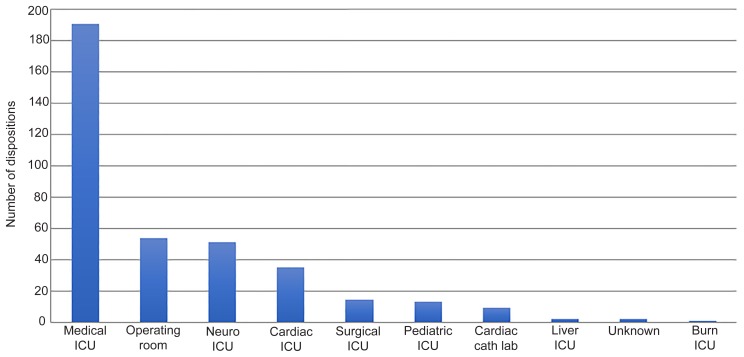
Final disposition location of mistriaged patients. *ICU*, intensive care unit.
